# Evaluation of copy-number variants as modifiers of breast and ovarian cancer risk for *BRCA1* pathogenic variant carriers

**DOI:** 10.1038/ejhg.2016.203

**Published:** 2017-02-01

**Authors:** Logan C Walker, Louise Marquart, John F Pearson, George A R Wiggins, Tracy A O'Mara, Michael T Parsons, BCFR, Daniel Barrowdale, Lesley McGuffog, Joe Dennis, Javier Benitez, Thomas P Slavin, Paolo Radice, Debra Frost, EMBRACE, Andrew K Godwin, Alfons Meindl, Rita Katharina Schmutzler, GEMO Study Collaborators, Claudine Isaacs, Beth N Peshkin, Trinidad Caldes, Frans BL Hogervorst, HEBON, Conxi Lazaro, Anna Jakubowska, Marco Montagna, KConFab Investigators, Xiaoqing Chen, Kenneth Offit, Peter J Hulick, Irene L Andrulis, Annika Lindblom, Robert L Nussbaum, Katherine L Nathanson, Georgia Chenevix-Trench, Antonis C Antoniou, Fergus J Couch, Amanda B Spurdle

**Affiliations:** 1 Department of Pathology, University of Otago, Christchurch, New Zealand; 2 Statistics Unit, QIMR Berghofer Medical Research Institute, Brisbane, Queensland, Australia; 3 Biostatistics and Computational Biology Unit, Department of the Dean, University of Otago, Christchurch, New Zealand; 4 Genetics and Computational Biology Division, QIMR Berghofer Medical Research Institute, Brisbane, Queensland, Australia; 5 Department of Epidemiology, Cancer Prevention Institute of California, Fremont, CA, USA; 6 Department of Public Health and Primary Care, Centre for Cancer Genetic Epidemiology, University of Cambridge, Strangeways Research Laboratory, Worts Causeway, Cambridge, UK; 7 Human Genetics Group, Spanish National Cancer Centre (CNIO), Madrid, Spain; 8 Department of Population Sciences Division of Clinical Cancer Genomics, City of Hope Clinical Cancer Genomics Community Research Network, Duarte, CA, USA; 9 Department of Preventive and Predictive Medicine, Unit of Molecular Bases of Genetic Risk and Genetic Testing, Fondazione IRCCS (Istituto Di Ricovero e Cura a Carattere Scientifico) Istituto Nazionale Tumori (INT), Milan, Italy; 10 Department of Pathology and Laboratory Medicine, University of Kansas Medical Center, Kansas City, KS, USA; 11 Department of Gynaecology and Obstetrics, Division of Tumor Genetics, Klinikum rechts der Isar, Technical University Munich, Munich, Germany; 12 Center for Hereditary Breast and Ovarian Cancer, Medical Faculty, University Hospital Cologne, Cologne, Germany; 13 Department of Tumour Biology, Institut Curie, Paris, France; 14 Institut Curie, INSERM, Paris, France; 15 Lombardi Comprehensive Cancer Center, Georgetown University, Washington, DC, USA; 16 Molecular Oncology Laboratory CIBERONC, Hospital Clinico San Carlos, IdISSC (El Instituto de Investigación Sanitaria del Hospital Clínico San Carlos), Madrid, Spain; 17 Family Cancer Clinic, Netherlands Cancer Institute, Amsterdam, The Netherlands; 18 The Hereditary Breast and Ovarian Cancer Research Group Netherlands (HEBON), Coordinating center: Netherlands Cancer Institute, Amsterdam, The Netherlands; 19 Molecular Diagnostic Unit, Hereditary Cancer Program, IDIBELL (Bellvitge Biomedical Research Institute), Catalan Institute of Oncology, Gran Via de l'Hospitalet, Barcelona, Spain; 20 Department of Genetics and Pathology, Pomeranian Medical University, Szczecin, Poland; 21 Immunology and Molecular Oncology Unit, Veneto Institute of Oncology IOV - IRCCS, Padua, Italy; 22 kConFab, Research Department, Peter MacCallum Cancer Centre, Melbourne, Australia; 23 The Sir Peter MacCallum Department of Oncology University of Melbourne, Parkville, Australia; 24 Department of Medicine, Cancer Biology and Genetics, Clinical Genetics Research Laboratory, Memorial Sloan-Kettering Cancer Center, New York, NY, USA; 25 Center for Medical Genetics, NorthShore University HealthSystem, Evanston, IL, USA; 26 Lunenfeld-Tanenbaum Research Institute, Mount Sinai Hospital, Toronto, Ontario, Canada; 27 Department of Clinical Genetics, Karolinska University Hospital, Stockholm, Sweden; 28 Department of Medicine and Institute for Human Genetics, University of California, San Francisco, CA, USA; 29 Department of Medicine and the Abramson Cancer Center, Perelman School of Medicine at the University of Pennsylvania, Philadelphia, PA, USA; 30 Department of Laboratory Medicine and Pathology, and Health Sciences Research, Mayo Clinic, Rochester, MN, USA

## Abstract

Genome-wide studies of patients carrying pathogenic variants (mutations) in *BRCA1* or *BRCA2* have reported strong associations between single-nucleotide polymorphisms (SNPs) and cancer risk. To conduct the first genome-wide association analysis of copy-number variants (CNVs) with breast or ovarian cancer risk in a cohort of 2500 *BRCA1* pathogenic variant carriers, CNV discovery was performed using multiple calling algorithms and Illumina 610k SNP array data from a previously published genome-wide association study. Our analysis, which focused on functionally disruptive genomic deletions overlapping gene regions, identified a number of loci associated with risk of breast or ovarian cancer for *BRCA1* pathogenic variant carriers. Despite only including putative deletions called by at least two or more algorithms, detection of selected CNVs by ancillary molecular technologies only confirmed 40% of predicted common (>1% allele frequency) variants. These include four loci that were associated (unadjusted *P*<0.05) with breast cancer (*GTF2H2*, *ZNF385B*, *NAALADL2* and *PSG5*), and two loci associated with ovarian cancer (*CYP2A7* and *OR2A1*). An interesting finding from this study was an association of a validated CNV deletion at the *CYP2A7* locus (19q13.2) with decreased ovarian cancer risk (relative risk=0.50, *P*=0.007). Genomic analysis found this deletion coincides with a region displaying strong regulatory potential in ovarian tissue, but not in breast epithelial cells. This study highlighted the need to verify CNVs *in vitro*, but also provides evidence that experimentally validated CNVs (with plausible biological consequences) can modify risk of breast or ovarian cancer in *BRCA1* pathogenic variant carriers.

## Introduction

Carriers of *BRCA1* pathogenic variants are at increased risk for developing breast cancer and/or ovarian cancer, but the precise level of these risks is uncertain. Estimates of the cumulative risks of breast and ovarian cancer by age 70 years for *BRCA1* pathogenic variant carriers range from 44% to 75% and 43 to 76%, respectively.^1^ Studies exploring the cause for the range in risk estimates have provided evidence that genetic factors have a key role in modifying cancer risks for carriers.^2^ The Consortium of Investigators of Modifiers of *BRCA1*/*BRCA2* (CIMBA) has facilitated a number of large studies, which have identified variants mapping to >20 loci that are associated with altered risk of breast or ovarian cancer in *BRCA1* pathogenic variant carriers.^3, 4, 5, 6^ The effect size associated with each variant identified to date has been relatively small (hazard ratio<1.5), and together they account for only a fraction of heritable variation in risk in *BRCA1* pathogenic variant-positive families.

Copy-number variants (CNVs) are estimated to cover 5–10%^7^ of the human genome, which is an order of magnitude greater than the number of base pairs (bp; ~15 Mbp; dbSNP Human Build 142) encompassed by the more commonly studied single-nucleotide polymorphisms (SNPs). Thus, based on base pair coverage, CNVs are responsible for the majority of genetic variability in human populations. CNVs have also been shown to partially overlap or fully encompass genes or regulatory sequences resulting in a range of biological changes, such as altered gene expression.^8^ Importantly, these inherited structural variants have a role in many complex diseases,^9^ and comprise a proportion of the mutation spectrum for known cancer syndromes, such as hereditary breast–ovarian cancer syndrome, Lynch syndrome and Li–Fraumeni syndrome.^10^ Moreover, recent genome-wide CNV studies have reported associations between a common deletion polymorphism overlapping *APOBEC3* and risk of both breast and ovarian cancer.^11, 12, 13^ Thus, other common and rare CNVs may similarly affect genes involved in cancer-related pathways. The contribution of germline CNVs to variable risk in individuals with deleterious *BRCA1* pathogenic variants is unknown.

In this paper, we conducted a large genome-wide CNV analysis of 2500 *BRCA1* pathogenic variant carriers, with or without breast and/or ovarian cancer, using a previously published SNP-based genome-wide association study.^14^ To maximize the sensitivity for CNV discovery, multiple CNV calling algorithms were applied to the data set. Analyses identified several putative CNVs overlapping gene regions associated with risk of breast or ovarian cancer for *BRCA1* pathogenic variant carriers and a requirement for validation in larger studies.

## Materials and methods

### Study population

A total of 2500 *BRCA1* pathogenic variant carriers was drawn from 20 centers from North America, Europe and Australia as reported previously.^14^ Eligibility criteria for study participants included the following: (1) female carriers of pathogenic *BRCA1* variants; (2) at least 18 years of age at recruitment; and (3) Caucasian self-reported ancestry. *BRCA1* pathogenic variant carriers selected for the study were stratified into two groups consisting of women diagnosed with invasive breast cancer when younger than 40-years old (*n*=1250) and women who had not developed breast cancer or who had developed a first ovarian cancer when 35 years of age or older (*n*=1250). All *BRCA1* pathogenic variants are listed in Supplementary Table S3 and deposited in the ClinVar database (Submission ID - SUB1994380; http://www.ncbi.nlm.nih.gov/clinvar/). All carriers were recruited for research studies using ethically approved protocols at host institutions.

### CNV detection and quality control

All DNA samples were genotyped with the Human610-Quad BeadChip (Illumina, Inc, San Diego, CA, USA) with ~610 000 markers (including ~20 000 non-polymorphic markers) for SNP and CNV analysis. Data for each array were normalized using GenomeStudio 2011.1 software (Illumina). Probe information including, genomic location, signal intensity (Norm R), allele frequency (Norm theta), Log R Ratios (LRRs), B allele frequencies (BAFs) for each sample was calculated and exported from GenomeStudio.

CNV calls were generated using four algorithms: PennCNV (version 2009 Aug27),^15^ QuantiSNP (v2.1),^16^ CNVPartition (v2.3.4, Illumina Inc.) and GNOSIS (a CNV detection algorithm within the CNV analysis package, CNVision, (http://sourceforge.net/projects/cnvision/files/). Quality control procedures were performed to remove poor quality array data (Supplementary Figure S3). Samples were excluded if they met the following criteria: PennCNV measures of log R ratio s.d.>0.28, BAF drift >0.01, waviness factor deviating from 0 by >0.05; QuantiSNP measures of BAF outliers >0.1, LogR outliers ≥0.1, BAF s.d. ≥0.2, LogR s.d. ≥0.4. A total of 2319 samples passed quality control steps and were assessed in the study. CNV calling results of all four algorithms were parsed and then merged using CNVision. To reduce false positives, CNV calls were excluded if ≥1000 kb in size, and/or were predicted by only one algorithm. Nine further CNVs called within the multi-histocompatibility complex on chromosome 6 were excluded from the study, as both a deletion and a duplication were predicted by two algorithms.

### Defining CNV regions that may contribute to modification of risk

To identify new genomic loci contributing to breast or ovarian cancer risk in *BRCA1* pathogenic variant carriers, common and rare deletions that overlapped gene regions were assessed using a genome-wide approach. Our study focused primarily on genomic deletions that overlapped gene regions for several reasons: (1) inter-individual analysis of CNVs is not straightforward as these variants do not typically occur in discrete genomic regions. The start and end coordinates of gene sequences were therefore used as a non-redundant approach to define CNV regions across the genome. (2) In contrast to duplications or copy-number gains, the genomic location of a deletion can be predicted from the array data. These data were not able to show the genomic location of a duplicated region, thus gene(s) or other functional genomic regions that are potentially disrupted by these structural events remain undetermined. (3) Whole or partial gene deletions are known to be potentially disruptive by causing haploinsufficiency or truncation of the expressed protein, and (4) common and rare CNVs that have previously been reported to be associated with breast and ovarian cancer risk have typically been deletion events.^11, 12, 17, 18^

We annotated 39 544 UCSC RefSeq (NCBI36/Hg18) transcripts using the SOURCE database^19^ and defined the genomic intervals for a total of 18 791 unique genes (Supplementary Figure S4). Thus, each gene interval encompassed the start and end of all corresponding alternate transcripts. CNVs and gene regions that were estimated to overlap by at least 1 bp were identified in a genome-wide scan using Intersect and Join tools from the Galaxy web server.^20, 21, 22^ All CNVs used for this study are deposited in the dbVar database (https://www.ncbi.nlm.nih.gov/dbvar) with the accession number nstd132.

### CNV validation

Accessible DNA samples from the study cohort were used to validate 29 putative deletion regions. All predicted common (>1% frequency) deletions found associated with breast or ovarian cancer risk were chosen for validation. Copy-number assessment was carried out using Nanostring nCounter Elements TagSets (NanoString Technologies, Inc.) and Taqman assays. Target-specific Nanostring probes for 10 CNV and 10 invariant genomic regions are listed in Supplementary Table S1. Twenty-two gene regions were assessed using Taqman assay, including one region (*GTF2H2*) also analysed by with a Nanostring TagSet. Custom primer and probe sequences are presented in Supplementary Table S2. For seven CNV loci, we used the pre-designed assays from Life Technologies (Supplementary Table S2).

### Statistical analysis

For the breast cancer risk association analysis, study participants (Supplementary Table S3) were classified at the age of the first breast cancer diagnosis or censored at ovarian cancer diagnosis or bilateral prophylactic mastectomy, whichever occurred first, or at the age of last observation. Only those diagnosed with breast cancer were considered as affected (*n*=1202 affected, *n*=1117 non-affected). Pathogenic variant carriers censored at their ovarian cancer diagnosis were considered to be unaffected in the breast cancer risk analyses. For ovarian cancer risk (*n*=357 affected, *n*=1962 non-affected), study participants were classified at the age of ovarian cancer diagnosis or censored at bilateral prophylactic oophorectomy, or age at last observation. Pathogenic variant carriers diagnosed with breast cancer were treated as unaffected at the age at breast cancer diagnosis. Analyses were carried out within a survival analysis framework. As *BRCA1* pathogenic variant carriers were not randomly sampled with respect to their disease status, analyses were based on the modeling the retrospective likelihood of observing the CNV conditional on the observed phenotype.^23^ Two separate models were fitted to evaluate associations between CNVs with breast cancer and ovarian cancer risk, and were assessed using the 1 d.f. score test statistic.^23^ Q-values for the discrete test statistics were calculated by filtering the *P*-values using the T-method^24^ with a critical threshold of 0.05, such that genes with total number of deletions of four or more were retained.

## Results

Genome-wide CNV analysis was performed on 2319 individuals with pathogenic *BRCA1* pathogenic variants, including 1202 breast cancer cases (1117 non-breast cancer affected) and 357 ovarian cancer cases (1962 non-ovarian cancer affected), using published genotype data from Illumina 610K SNP arrays.^14^ A total of 60 893 CNVs were called across the study participants using four different algorithms (PennCNV, QuantiSNP, GNOSIS and CNVPartition) that passed the data quality threshold (see Methods and Materials). Of these, 89% and 94% CNVs were predicted by PennCNV and QuantiSNP, respectively, compared with a lower prediction rate from GNOSIS (35%) and CNVPartition (42% Supplementary Table S4). The average number of CNVs observed per individual was 26.3 (range 4–203) that ranged in size from 314 to 999 990 bases.

A total of 21 013 CNVs were predicted to overlap at least one of 5848 different RefSeq genes across the study cohort. The average number of CNVs overlapping genes per individual was 9.1 (range 1–107). Deletions overlapping genes were detected approximately three times as often than duplications (6.8 *versus* 2.2, respectively). Interrogating the CNV calls from at least two algorithms revealed a deletion overlapping the *BRCA1* gene in 14 study participants (Supplementary Figure S1). In each case, the deletion was confirmed by agreement with the results from the diagnostic *BRCA1* germline genetic tests, supporting the use of two or more algorithms to reduce the possibility of artifactual CNV calling and false discoveries. However, CNV calling was unable to identify *BRCA1* deletions overlapping five or more probes in eight pathogenic variant carriers that had previously been identified by diagnostic testing. These results therefore show a 100% detection specificity and a 64% detection sensitivity for CNV calls across the *BRCA1* gene region. Two algorithms (PennCNV and QuantiSNP) dominated the CNV calling in this region, with PennCNV alone detecting a deletion in 14 cases (Supplementary Figure S1). No further *BRCA1* deletions were identified using just one algorithm (data not shown).

Analysis of 5848 putative deletions delineated by gene regions identified a total of 52 loci associated (at unadjusted *P*<0.05) with breast cancer risk (Supplementary Table S6), and 72 CNV loci associated with ovarian cancer risk for *BRCA1* pathogenic variant carriers (Supplementary Table S6). The top predicted CNV regions associated with risk included *FGFR1OP2* (RR=0.20, *P*=5 × 10^−4^) and *PABPC4L* (RR=0.22, *P*=0.006) for breast and ovarian cancer, respectively. Eight loci (*PABPC4L*, *APBA2*, *FAM189A1*, *FUT7*, *ENTPD2*, *NPDC1*, *C9orf139* and *L1CAM*) were associated with risks for both breast cancer and ovarian cancer (*P*<0.05).

SNP arrays are well known for low accuracy when assessing CNVs, compared with other platforms such as bacterial artificial chromosome array and oligonucleotide arrays.^25^ We therefore attempted to validate CNV regions using Nanostring technology, qPCR and data from the recently published Human CNV Map.^7^ Twenty-nine predicted CNV loci were selected for validation including the most common deletions (>1% frequency) found to be associated with breast or ovarian cancer risk in the *BRCA1* pathogenic variant carrier cohort. Eight of these 29 (28%) CNV loci were confirmed by qPCR and/or Nanostring analysis, including four loci that were associated with breast cancer (*GTF2H2*, *ZNF385B*, *NAALADL2* and *PSG5*) and two loci that were associated with ovarian cancer (*CYP2A7* and *OR2A1*; Table 1). Nanostring analysis of eight putative CNV loci from Table 1 (*ZNF385B*, *CALCRL*, *TFPI*, *GTF2H2*, *SLCO1B1 FGFR1OP2*, *TM7SF3* and *ALX1*) in 48 study samples only found seven deletions not identified by the calling algorithms, suggesting a low false negative rate (2% (7_Nanostring calls_/352_Negative bioinformatic calls_); Supplementary Table S5). The strongest association with a validated deletion was observed for ovarian cancer, detected in 75/1962 (3.8%) unaffected carriers and 4/357 (1.1%) affected carriers (RR=0.50, *P*=7 × 10^−3^) overlapping the *CYP2A7* locus (19q13.2; Supplementary Figure S2).

**Figure 1:**
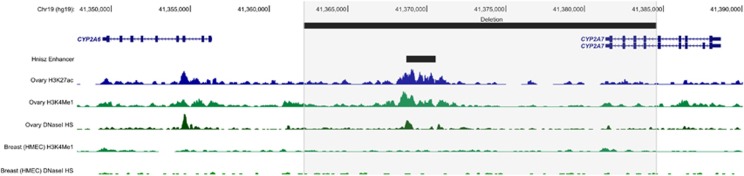
Genomic landscape at the region containing the CNV deletion overlapping *CYP2A7*. The location of the CNV deletion and the enhancer predicted by Hnisz *et al*^28^ to affect *EGLN2* are shown by black bars. Histone modifications associated with enhancer elements (H3K27Ac and H3K4Me1) and DNAseI hypersensitivity sites (HSs) for normal ovarian tissue and normal breast epithelial cells (HMECs) from Roadmap Epigenomics Consortium and ENCODE are depicted by histogram tracks.

To assess the functional relevance of the validated CNV deletion overlapping the *CYP2A7* locus, the genomic landscape at this region was investigated using publicly available genomic data from ENCODE^26^ and the Roadmap Epigenomics Consortium^27^ (Figure 1). Examining data generated from normal ovarian tissue, the CNV deletion coincided with enhancer-specific histone modifications (acetylation of H3 lysine 27 (H3K27Ac) and mono-methylation of H3 lysine 4 (H3K4Me1)) and DNaseI hypersensitivity sites representative of open chromatin. By contrast, there was no evidence for these chromatin features in normal breast epithelial (HMEC) cells. Cross-reference to super-enhancers annotated in the study by Hnisz *et al*,^28^ found the CNV deletion overlapped an enhancer, found in ovary tissue, predicted to affect the expression of *EGLN2*, located ~67 kb downstream of *CYP2A7*.

Zarrei *et al*^7^ recently published a Human CNV Map constructed from multiple studies in the Database of Genomic Variants by applying a clustering algorithm to define ~27 000 CNV regions with high stringency. Comparing this stringent map with validated CNVs from this study revealed a strong consensus. All eight CNV loci validated in *BRCA1* pathogenic variant carriers were present in the published CNV Map, and only one CNV (*CNTNAP3B*) that was not verified in our data was present in the CNV Map (Table 1). Using the published Human CNV Map to support the existence of putative CNVs from this association study identified deletions at nine of 52 gene loci (17%) that are associated with breast cancer risk (Supplementary Table S6), and 13 of 72 (18%) gene loci associated with ovarian cancer risk (Supplementary Table S7). With the exception of the *CYP2A7* locus (*P*=0.007), all validated CNV regions returned a modest association (*P*-values ranged from 0.01 to 0.049) for ovarian or breast cancer risk. Validated CNVs ranged in allele frequency from 0.2 to 7.8%.

## Discussion

Compared with SNPs, the contribution of CNVs to genetic variability and breast and/or ovarian cancer risk is relatively unknown. This is the first genome-wide CNV association study of *BRCA1* pathogenic variant carriers to identify CNVs that are associated with breast and/or ovarian cancer risk, and the first implementation of the retrospective likelihood to CNV data. Our study used multiple CNV calling algorithms with the aim of increasing the sensitivity and specificity of CNV detection. Initial assessment of known deletions overlapping the *BRCA1* gene indicated 100% detection specificity and 64% detection sensitivity. This assessment also showed that all 14 CNVs identified at *BRCA1* were called by two or more calling algorithms, setting the calling criteria for the remainder of the study. However, validation of 29 predicted CNVs throughout the genome confirmed <30% of predicted deletions, highlighting a large number of false variant calls. None of the nine rare variants (<1% allele frequency) chosen for validation was verified by qPCR or Nanostring. However, CNV calling correctly predicted 40% (8/20) deletions we tested which ranged in allele frequency from 1.2 (*OR2A1* locus) to 8.2% (*ZNF385B* locus). These results confirm other published reports that indicate array-based CNV data can be unreliable without further validation using ancillary technologies, such as qPCR.^25^ The accuracy may be increased by employing more stringent criteria but likely at the expense of detection sensitivity. For example, a larger number of probe markers could be used to generate a CNV call, but this approach will also reduce the spatial resolution of the array and sensitivity. PennCNV algorithm called ~90% of variants assessed in this study including all the deletions that were detected across *BRCA1* in 14 cases. These data suggest that the combination of four algorithms for generating putative CNV information may not have been a vast improvement over using PennCNV alone.

Our study focused on genomic deletions that overlapped gene regions, as this approach provided functionally important genomic regions for comparing CNV calls. A notable finding was an association of a CNV deletion at the *CYP2A7* locus (19q13.2) with decreased ovarian cancer risk (RR=0.50, *P*=0.007). To our knowledge, this locus has not previously been associated with cancer risk from SNP-based or CNV-based genome-wide association studies and requires further investigation. *CYP2A7* encodes a member of the cytochrome P450 superfamily of enzymes, although the substrate(s) for this gene have not yet been determined. The deletion variant in this region may also affect the regulation of a nearby gene *CYP2A6*,^29^ which is known to have a key role in the metabolism of a number of substrates including nicotine, coumarin and valproic acid.^30^ Interestingly, a deletion at the *CYP2A6* locus has been found to be associated with decreased risk of lung cancer in Asian smokers,^31^ which is comparable to our finding that *CYP2A7* deletions were more frequent in non-affected high-risk *BRCA1* pathogenic variant carriers compared with those with ovarian cancer (MAF—3.8% *versus* 1.1%). Examining published data from The Cancer Genome Atlas showed that ~40% of high-grade serous ovarian tumors, including 6% *BRCA1* pathogenic variant carriers, exhibited somatic hemizygous deletions overlapping *CYP2A7*.^32^ Moreover, these deletions correlated with a reduced expression level compared with copy neutral *CY2A7* (Supplementary Figure S5). These data indicate that, although a germline deletion of *CYP2A7* may protect against initiation of ovarian cancer in the context of a *BRCA1* germline pathogenic variant, somatic deletions of *CYP2A7* may be important for the ovarian cancer development or progression.

Analysis of chromatin features from normal ovary tissue at the *CYP2A7* genomic region shows that the CNV deletion coincides with chromatin marks consistent with an enhancer element. Interestingly, there was no evidence of similar features in normal breast epithelial cells, suggesting a tissue-specific feature. These results are concordant with the association of this CNV deletion with ovarian, and not breast, cancer risk in this cohort. Cross-reference of this region to the catalog of enhancers compiled by Hnisz *et al*^28^ found the CNV deletion overlaps a putative enhancer in ovarian tissue. This enhancer is predicted to affect expression of *EGLN2*, which encodes an enzyme involved in oxygen homeostasis. Further biological experiments are required to delineate the mechanism underlying the observed association between the CNV deletion and ovarian cancer risk. Importantly, although we prioritized CNVs for analysis based on overlap with coding genes, our findings suggest that intergenic CNVs could confer risk by altering regulatory elements. Therefore, future analyses integrating chromatin features into the CNV selection process could identify other CNVs, missed in this analysis, associated with cancer risk.

Confirmed deletions overlapping a total of nine gene loci were found associated with breast cancer risk, and a total of 13 gene loci associated with ovarian cancer risk in *BRCA1* pathogenic variant carriers (Supplementary Table S5). GTF2H2 (5q13.2) is a transcription factor with a role in the nucleotide excision repair (NER) pathway,^33^ a DNA repair pathway that is disrupted in *BRCA1*-associated breast cancers. Deletions overlapping *GTF2H2* are associated with decreased risk of breast cancer, suggesting that disruption of NER may be protective against the biological consequences of a *BRCA1* pathogenic variant. The potential biological effect of the remaining deletions is unclear.

Genetic associations identified by this study included rare (<1% MAF) and polymorphic (>1% MAF) deletions that occurred at relatively low frequency (<10%) within the study cohort. Notably, no deletion polymorphism was observed overlapping the *APOBEC3* locus, which has previously been associated with risk of both breast and ovarian cancer.^11, 12, 13^ This might be expected as the Illumina 610k array contains only two probes across the CNV region located between the fifth exon of *APOBEC3A* and the eighth exon of *APOBEC3B* so the variant is unlikely to be detected.^12^ Although this study identifies CNVs in *BRCA1* pathogenic variant carriers, the low frequency of CNVs (all <10% in this study) and sample size limits the power to detect association in this study, in particular no associations reported here are significant after controlling for a false discovery rate of 0.05.^34^ Replication of CNVs identified by this study using larger data sets will be required to verify these associations. Moreover, larger cohort sizes will facilitate more detailed analyses to be performed, such as competing risks analyses to evaluate the associations with breast and ovarian cancer risks simultaneously. Importantly, genotyping data currently being derived by the large Oncoarray Network containing DNA samples from ~20 000 *BRCA1* pathogenic variant carriers (http://epi.grants.cancer.gov/oncoarray/) will enable additional genome-wide CNV analysis and further assessment of candidate gene regions identified by this study.

## Figures and Tables

**Table 1:** Validation results from predicted deletions at gene loci for breast cancer risk, ovarian cancer risk and test CNVs

				*Validation*		
*Gene locus*	*MAF (array data)*	P*-value*	*Relative risk (95% CI)^a^*	*Nanostring*	*qPCR*	*Present on CNV map*^b^
*Breast cancer risk*						
* FGFR1OP2*	0.6%	0.0005	0.2 (0.1–0.38)	0% (0/3)	—	No
* TM7SF3*	0.5%	0.004	0.2 (0.09–0.45)	0% (0/3)	—	No
* CALCRL*	0.4%	0.006	4.13 (1.29–13.2)	0% (0/4)	—	No
* TFPI*	0.4%	0.006	4.13 (1.29–13.2)	0% (0/4)	—	No
* **GTF2H2***	3.4%	0.01	0.64 (0.45–0.91)	33% (2/6)	66% (2/3)	**Yes**
* CPSF1*	1.1%	0.02	2.03 (1.09–3.81)	—	0% (0/3)	No
* SLCO1B1*	0.9%	0.03	0.42 (0.23–0.78)	0% (0/4)	—	No
* ALX1*	0.2%	0.03	0.23 (0.06–0.94)	0% (0/3)	—	No
* GRIN1*	1.1%	0.03	0.51 (0.28–0.9)	—	0% (0/3)	No
* **ZNF385B***	8.2%	0.04	0.79 (0.62–1.01)	100% (12/12)	—	**Yes**
* ABR*	1.0%	0.04	1.85 (0.96–3.56)	—	0% (0/1)	No^c^
* **NAALADL2***	7.80%	0.05	1.25 (0.96–1.62)	—	100% (3/3)	**Yes**
* **PSG5***	3.20%	0.05	0.7 (0.48–1.03)	—	100% (2/2)	**Yes**
* RER1*	1.60%	0.05	1.69 (1.01–2.84)	—	0% (0/2)	No

*Ovarian cancer risk*						
* **CYP2A7***	3.4%	0.007	0.5 (0.2–1.27)	—	100% (5/5)	**Yes**
* PTPRD*	1.30%	0.01	0.4 (0.1–1.56)	—	0% (0/3)	No^c^
* DACH1*	12.9%	0.02	1.57 (1.11–2.23)	—	0% (0/9)	No
* UGT2A1*	0.2%	0.03	0.28 (0–68.3)	—	0% (0/1)	No
* C9orf140*	1.0%	0.03	3.59 (1.52–8.46)	—	0% (0/2)	No^c^
* RAB43*	1.90%	0.03	0.44 (0.2–1)	—	0% (0/3)	No
* *UAP1L1	1.00%	0.03	3.63 (1.53–8.53)	—	0% (0/3)	No
* PTPRK*	0.3%	0.04	0.45 (0.02–12.25)	—	0% (0/2)	No
* APRT*	1.1%	0.04	3.04 (1.38–6.72)	—	0% (0/2)	No
* PRKG1*	2.20%	0.05	0.49 (0.21–1.16)	—	0% (0/3)	No
* **OR2A1***	1.20%	0.05	3.97 (1.7–9.29)	—	100% (3/3)	**Yes**

*Test CNVs*^d^						
* **EPHA3***	6.8%	0.14	1.19 (0.9–1.56)	—	100% (5/5)	**Yes**
* CNTNAP3B*	3.5%	0.79	1.05 (0.7–1.56)	0% (0/3)	—	**Yes**
* **NAIP***	2.4%	0.11	0.71 (0.46–1.1)	—	100% (4/4)	**Yes**
* ELP4*	0.9%	0.63	0.84 (0.43–1.65)	—	0% (0/3)	No

Abbreviations: CI, confidence interval; CNV, copy-number variant; MAF, minor allele frequency; qPCR, quantitative PCR.

a Approximate relative risk values were calculated using the Score Test^23^.

b A copy-number variation map of the human genome (Zarrei *et al*).^7^

c CNV regions from the Zarrei *et al*^7^ map overlap the gene of interest but not the CNVs called by this study.

d Breast cancer risk for four test CNVs with MAF ranging from 0.9 (rare) to 6.8% (common) selected for technical validation.

Loci that were validated by Nanostring and/or qPCR assays are shown in bold.
