# Correction: Evaluation of copy-number variants as modifiers of breast and ovarian cancer risk for BRCA1 pathogenic variant carriers

**DOI:** 10.1038/s41431-018-0216-1

**Published:** 2018-08-22

**Authors:** Logan C. Walker, Louise Marquart, John F. Pearson, George A. R. Wiggins, Tracy A. O’Mara, Michael T. Parsons, BCFR, Daniel Barrowdale, Lesley McGuffog, Joe Dennis, Javier Benitez, Thomas P. Slavin, Paolo Radice, Debra Frost, EMBRACE, Andrew K. Godwin, Alfons Meindl, Rita Katharina Schmutzler, GEMO Study Collaborators, Claudine Isaacs, Beth N. Peshkin, Trinidad Caldes, Frans B. L. Hogervorst, HEBON, Conxi Lazaro, Anna Jakubowska, Marco Montagna, KConFab Investigators, Xiaoqing Chen, Kenneth Offit, Peter J. Hulick, Irene L. Andrulis, Annika Lindblom, Robert L. Nussbaum, Katherine L. Nathanson, Georgia Chenevix-Trench, Antonis C. Antoniou, Fergus J. Couch, Amanda B. Spurdle

**Affiliations:** 1 Department of Pathology, University of Otago, Christchurch, New Zealand; 2 Statistics Unit, QIMR Berghofer Medical Research Institute, Brisbane, Queensland Australia; 3 Biostatistics and Computational Biology Unit, Department of the Dean, University of Otago, Christchurch, New Zealand; 4 Genetics and Computational Biology Division, QIMR Berghofer Medical Research Institute, Brisbane, Queensland Australia; 5 Department of Epidemiology, Cancer Prevention Institute of California, Fremont, CA USA; 6 Department of Public Health and Primary Care, Centre for Cancer Genetic Epidemiology, University of Cambridge, Strangeways Research Laboratory, Worts Causeway, Cambridge, UK; 7 Human Genetics Group, Spanish National Cancer Centre (CNIO), Madrid, Spain; 8 Department of Population Sciences Division of Clinical Cancer Genomics, City of Hope Clinical Cancer Genomics Community Research Network, Duarte, CA USA; 9 Department of Preventive and Predictive Medicine, Unit of Molecular Bases of Genetic Risk and Genetic Testing, Fondazione IRCCS (Istituto Di Ricovero e Cura a Carattere Scientifico) Istituto Nazionale Tumori (INT), Milan, Italy; 10 Department of Pathology and Laboratory Medicine, University of Kansas Medical Center, Kansas City, KS USA; 11 Department of Gynaecology and Obstetrics, Division of Tumor Genetics, Klinikum rechts der Isar, Technical University Munich, Munich, Germany; 12 Center for Hereditary Breast and Ovarian Cancer, Medical Faculty, University Hospital Cologne, Cologne, Germany; 13 Department of Tumour Biology, Institut Curie, Paris, France; 14 Institut Curie, INSERM, Paris, France; 15 Lombardi Comprehensive Cancer Center, Georgetown University, Washington, USA; 16 Molecular Oncology Laboratory CIBERONC, Hospital Clinico San Carlos, IdISSC (El Instituto de Investigación Sanitaria del Hospital Clínico San Carlos), Madrid, Spain; 17 Family Cancer Clinic, Netherlands Cancer Institute, Amsterdam, The Netherlands; 18 The Hereditary Breast and Ovarian Cancer Research Group Netherlands (HEBON), Coordinating center: Netherlands Cancer Institute, Amsterdam, The Netherlands; 19 Molecular Diagnostic Unit, Hereditary Cancer Program, IDIBELL (Bellvitge Biomedical Research Institute), Catalan Institute of Oncology, Gran Via de l’Hospitalet, Barcelona, Spain; 20 Department of Genetics and Pathology, Pomeranian Medical University, Szczecin, Poland; 21 Immunology and Molecular Oncology Unit, Veneto Institute of Oncology IOV - IRCCS, Padua, Italy; 22 kConFab, Research Department, Peter MacCallum Cancer Centre, Melbourne, Australia; 23 The Sir Peter MacCallum Department of Oncology University of Melbourne, Parkville, Australia; 24 Department of Medicine, Cancer Biology and Genetics, Clinical Genetics Research Laboratory, Memorial Sloan-Kettering Cancer Center, New York, NY USA; 25 Center for Medical Genetics, NorthShore University HealthSystem, Evanston, IL USA; 26 Lunenfeld-Tanenbaum Research Institute, Mount Sinai Hospital, Toronto, Ontario, Canada; 27 Department of Clinical Genetics, Karolinska University Hospital, Stockholm, Sweden; 28 Department of Medicine and Institute for Human Genetics, University of California, San Francisco, CA USA; 29 Department of Medicine and the Abramson Cancer Center, Perelman School of Medicine at the University of Pennsylvania, Philadelphia, PA USA; 30 Department of Laboratory Medicine and Pathology, and Health Sciences Research, Mayo Clinic, Rochester, MN USA

Correction to: *European Journal of Human Genetics* 10.1038/ejhg.2016.203; Article published online 1 February 2017

This Article was originally published under a CC BY-NC-SA 4.0 license, but has now been made available under a CC BY 4.0 license. The PDF and HTML versions of the Article have been modified accordingly.

