# Supplementary material for: Evaluation of copy-number variants as modifiers of breast and ovarian cancer risk for BRCA1 pathogenic variant carriers
Source: Eur J Hum Genet. 2017 Feb 1;25(4):432–8. doi: 10.1038/ejhg.2016.203 (PMC5386423; doi:10.1038/ejhg.2016.203)
Supplement: Supplementary Information [file ejhg2016203x1.docx]

**Evaluation of copy number variants as modifiers of breast and ovarian cancer risk for *BRCA1* pathogenic variant carriers**

Logan C. Walker, Louise Marquart, John F. Pearson, George AR Wiggins, Tracy A O’Mara, BCFR, Daniel Barrowdale, Lesley McGuffog, Joe Dennis, Javier Benitez, Thomas P. Slavin, Paolo Radice, Debra Frost, EMBRACE, Andrew K. Godwin, Alfons Meindl, Rita Katharina Schmutzler, GEMO Study Collaborators, Claudine Isaacs, Beth N. Peshkin, Trinidad Caldes, Frans B.L. Hogervorst, HEBON, Conxi Lazaro, Anna Jakubowska, Marco Montagna, KConFab Investigators, Xiaoqing Chen, Kenneth Offit, Peter J. Hulick, Irene L. Andrulis, Annika Lindblom, Robert L. Nussbaum, Katherine L. Nathanson, Georgia Chenevix-Trench, Antonis C. Antoniou, Fergus J. Couch, Amanda B. Spurdle

**Supplementary Information:**

Contents

[SUPPLEMENTARY TABLES AND FIGURES 3](#_Toc453052943)

[Supplementary Figure S1. Detection of known *BRCA1* deletions using four different algorithms. 3](#_Toc453052944)

[Supplementary Figure S2. Deletion variants identified in the *CYP2A7* region associated with ovarian cancer. 4](#_Toc453052945)

[Supplementary Figure S3. Study design for CNV discovery, quality control and analysis 5](#_Toc453052946)

[Supplementary Figure S4. Work flow for identifying genomic coordinates for RefSeq gene boundaries 6](#_Toc453052947)

[Supplementary Figure S5. Expression level and copy number status of CYP2A7 in ovarian carcinomas from The Caner Genome Atlas data (http://www.cbioportal.org/). 7](#_Toc453052948)

[Supplementary Table S1. nCounter Elements design details for NanoString assays 8](#_Toc453052949)

[Supplementary Table S2. Taqman assay primer and probe sequences, or proprietary design 9](#_Toc453052950)

[Supplementary Table S3. Genotype and phenotype of study cohort 9](#_Toc453052951)

[Supplementary Table S4. Number of CNVs of each type by calling algorithm 9](#_Toc453052952)

[Supplementary Table S5. Nanostring validation results of predicted copy number deletions associated with breast cancer risk across eight gene loci in 48 study samples. 10](#_Toc453052953)

[Supplementary Table S6. Associations with breast cancer risk for deletion variants overlapping gene regions. Genes that overlap with the Human CNV Map are shown in bold. 11](#_Toc453052954)

[Supplementary Table S7. Associations with ovarian cancer risk for deletion variants overlapping gene regions. Genes that overlap with the Human CNV Map are shown in bold. 12](#_Toc453052955)

[REFERENCES 13](#_Toc453052956)

## SUPPLEMENTARY TABLES AND FIGURES

# Supplementary Figure S1. Detection of known *BRCA1* deletions using four different algorithms.

UCSC Genome Browser image of the region containing *BRCA1*. Black Bars indicated the CNVs identified in 14 different *BRCA1* mutation carriers by at least two CNV calling algorithms (G – GNOSIS; C – CNVPartition; P – PennCNV; Q – QuantiSNP). CNV calling results from each algorithm are shown (1 – deletion; 2 – no copy number change).


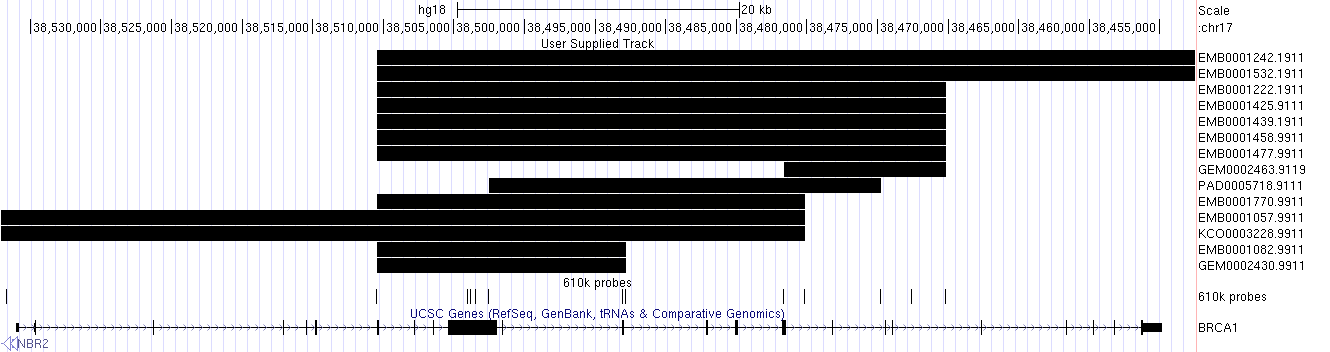


| **Sample ID** | **BIC nomenclature for *BRCA1* pathogenic variant** | **HGVS Genomic nomenclature for *BRCA1* pathogenic variant** | **# overlapping probes** |  | **Sample ID** | **BIC nomenclature for *BRCA1* pathogenic variant** | **HGVS Genomic nomenclature for *BRCA1* pathogenic variant** | **# overlapping probes** |
| --- | --- | --- | --- | --- | --- | --- | --- | --- |
| 1242 | del exons 2-24 | c.-19-?_5592+?del | 12 |  | 1446 | exon14-20del26kb | c.4358-?_5277+?del | 5 |
| 1532 | del exons 3-19 | c.81-?_5193+?del | 10 |  | 1530 | del exons 3-19 | c.81-?_5193+?del | 10 |
| 1222 | del exons 1-23 | c.-200-?_5467+?del | 12 |  | 1537 | del exons 1-12 | c.-200-?_4185+?del | 5 |
| 1425 | del exons1-21 | c.-200-?_5332+?del | 12 |  | 1538 | del exons 1-12 | c.-200-?_4185+?del | 5 |
| 1439 | del exons 1-23 | c.-200-?_5467+?del | 12 |  | 1689 | del exons 1-17 | c.-200-?_4185+?del | 8 |
| 1458 | del exons 1-23 | c.-200-?_5467+?del | 12 |  | 2413 | del exons 8-13 | c.442-?_4357+?del | 5 |
| 1477 | del exons1-21 | c.-200-?_5332+?del | 12 |  | 3382 | del exon14-20 | c.4358-?_5277+?del | 5 |
| 2463 | del exons 16-23 | c.4676-?_5467+?del | 5 |  | 7236 | del exon14-20 | c.4358-?_5277+?del | 5 |
| 5718 | del exons 9-19 | c.548-2247_5194-6021del36381 | 11 |  | Abbreviations: BIC, Breast Cancer Information Core (<https://research.nhgri.nih.gov/bic/>); HGVS, Human Genome Variation Society; ID, Identification.  Coding DNA reference sequence from genomic Refseq NG_005905.2 covering *BRCA1* transcript NM_007294.3. Exon boundaries from GenBank U14680.1, that is, exon 4 is missing due to a correction made after the initial description of *BRCA1*. | | | |
| 1770 | del exons 1-17 | c.-200-?_5074+?del | 8 |  |  |  |  |  |
| 1057 | del exons 1-17 | c.-200-?_5074+?del | 8 |  |  |  |  |  |
| 3228 | del exons 1-17 | c.-200-?_5074+?del | 8 |  |  |  |  |  |
| 1082 | del exons 8-13 | c.442-?_4357+?del | 5 |  |  |  |  |  |
| 2430 | del exons 8-13 | c.442-?_4357+?del | 5 |  |  |  |  |  |

Supplementary Figure S2. Deletion variants identified in the *CYP2A7* region associated with ovarian cancer. UCSC Genome Browser image of the region containing *CYP2A7*. Seventy four deletions (blue blocks) were identified in non-breast cancer affected females compared to fours deletions (red blocks) in ovarian cancer affected females.


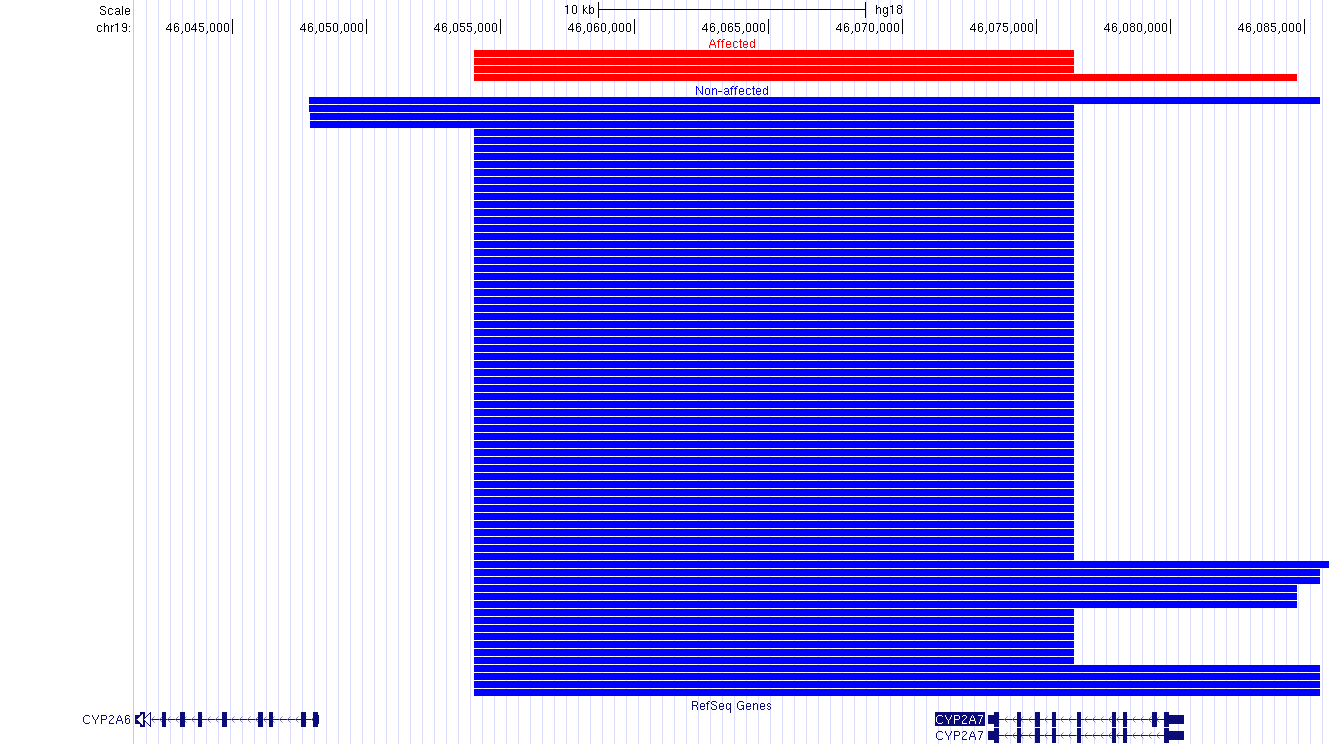


# Supplementary Figure S3. Study design for CNV discovery, quality control and analysis

**2500 *BRCA1* pathogenic variant carriers^1^**

(1250 breast and/or ovarian cancer affected, 1250 non-affected)

Illumina Human610k BeadChip

**2378 samples**

122 samples (poor or no genotyping data, non-European ancestry, sex errors, sample duplication)

**2319 samples**

(1202 affected, 1117 non-affected)

QC

59 samples (poor quality PennCNV and/or QuantiSNP calls)

CNV calling and QC

Analysis of CNVs by gene-centric genomic regions

CNVs (<2 algorithms, >1Mb)

# Supplementary Figure S4. Work flow for identifying genomic coordinates for RefSeq gene boundaries

# Supplementary Figure S5. Expression level and copy number status of *CYP2A7* in ovarian serous cystadenocarcinoma based upon data generated by The Cancer Genome Atlas (TCGA) Research Network: http://cancergenome.nih.gov/.

| ***CYP2A7* copy number status** | **Sample size** | **Mean *CYP2A7* mRNA z-score** | **Standard deviation** | **P-value^1^** |
| --- | --- | --- | --- | --- |
| Deletion | 205 | -0.164 | 0.764 | 0.049 |
| Copy neutral | 175 | 0.015 | 0.999 |  |
| ^1^ t-test – two-tailed, equal variance | | | | |

# Supplementary Table S1. nCounter Elements design details for NanoString assays

| **Region Name** | **Genomic Coordinates** | **Target Sequence** | **A Tm** | **B Tm** |
| --- | --- | --- | --- | --- |
| INVCONTROL-1 | chr1:97239055-97239154 | AGATAGCAACAACTCATGGGGTAATCAGATGGACTGAATGAGATAACACGTTAAATGTGAACACAGCAACTTACCTGGAGTCGGTACACATCAAATGTTA | 84 | 83 |
| INVCONTROL-2 | chr2:137221998-137222092 | TTTTGATGAAAGTATTGTCTTAAATCTGCAAACGCAAAATTGTGTGATGGCTTATGCATCAGAGGGTTGGTTCTAAAGCACTTTGTGGTCAACTCCAAGG | 81 | 84 |
| INVCONTROL-5 | chr5:71261236-71261335 | CAGATCTCCTGTGATTCTTCTGTATATCTGGAGAAGATGATCAAACACCACTACCGGTTGAGGATGTGATATATGGAGATTCGGGAATCTGAGGGTTTAT | 82 | 84 |
| INVCONTROL-7 | chr7:41541153-41541252 | TGATTTGGTTGCTGTTGGCATTATGCTTTATTTTAAGAGGGCTCCACTAAAAATCAGGGAGGTGGTAACTCTGTCCAGAACACCAAAATTTATAATTTCG | 84 | 83 |
| INVCONTROL-10 | chr10:76411622-76411718 | ACTCCAAGAAGTGTGGATGGTTTCATCCTCCAGCAAATGAAATTTACCGAAGGAAAGACCTTTCAGTATTTGAGGTAAGCGCGTGTAAATAAAAAAATTC | 84 | 81 |
| INVCONTROL-16 | chr16:11026537-11026636 | GATGACTATATTCACCCTGTGGTTTTCTTTTGACCCCTTGATTTACAACTGACTTAGCGTCTGACCTCTTTCCTAACATCAGAACTGAAATCAGTCAAAT | 83 | 83 |
| INVCONTROL-17 | chr17:60617472-60617571 | GATGTGACTAATCATGTATTCTGACTGTGTAAGACTCAAGTCATGGTACTAATCGGGACTGAAGATGCTGTTTTGTTTAGTGCCTTCCTTCAATATGTTT | 81 | 84 |
| INVCONTROL-19 | chr19:38818850-38818949 | AAAAACCTCTTGACGCTTTCTCCAAAATTGATCTGAAGACTTGGCAGATTTGAGGAATCATTCAGTAACGTTTTACGAGTCGATGCTAATTTGATGCGGC | 84 | 85 |
| INVCONTROL-20 | chr20:10504898-10504992 | CCTTTGGTACGATAGACATTTATACTGTCTCTAAGTGAATTTGCCACAGATTCAGAGTGAGGTGCTTATTTGTGAAGCATTAAGAATCCCTGGGCTGTTC | 82 | 84 |
| INVCONTROL-22 | chr22:42509372-42509471 | TATTTGCTCATGAATACTTACTTGAAGAAGATCTGAACTTATTTGGGGAACCATTCCTTGAATATACTCTACACGGTGAAGAATGGGGTCTTGAATGTGT | 80 | 84 |
| *CALCRL* | chr2:187957029-187957128 | AGCTGTTATCTTCCTATCCTTCCTTTGCCTCCTCATTGTAGTATTTCTTTTTTCTAGCAGGAAGTTGAATTTAGTAAAATCAAAATGACATTCCCTCACT | 82 | 80 |
| *GTF2H2* | chr5:70389792-70389891 | AAAAAAAAAAGAAGTAATAGAAAACTGCTGGTAACTCGGGGTGGGTAAAGTGAGAGGAAAATATTGACCTTATATTCCTACCTTGGAGGAACCATCCTTC | 82 | 83 |
| *TFPI* | chr2:188068557-188068651 | GCTTGGGAGCATACAACTAAACATCTTACAGAGCCACAGAGGAGAGAAGTTCACTCTTTTCACCACATGGAAGTTTTCCCACAGGCCAAGAGCACCATGA | 86 | 87 |
| *TM7SF3* | chr12:27019857-27019956 | AGCTGTGGAAGAGTGGAAGAGTTTAGGCATCATCCATGTTAGAACAAAAATAGTTTATAACTCTCCACTGGCTTCAATCACAGGCAATCACATCTGCTCC | 84 | 85 |
| *ZNF385B* | chr2:180126562-180126661 | TAAAATCACTCAGTATGTGGCAGAGCCTGGGTCCAAACTCCTGTCCATCTGACTTTAAAGCCCATGTCATCATTCCCTAAAGTAGGTTATATGTAAAGCT | 87 | 81 |
| *ALX1* | chr12:84209900-84209999 | AGAATTCCTACTACTTATTAAGTTCTGTTTTGTACTAGGCAATGCCCTAAGTAATTTGCATAAACAAGTTTGTTTAACATTCACAAGCATCGTATGAGCT | 80 | 81 |
| *CNTNAP3B* | chr9:43639950-43640049 | ATTAGTAAGATGGCAGTGTCAGAATCCTTGAGAAAATAATAACCAGAACATCTTAGAATTTATTGTGCCCAAGGAAGGAGCCCTGTCAATAGACAAATGT | 81 | 84 |
| *FGFR1OP2* | chr12:27004404-27004503 | AAAACTTCTAGTTAAGGGCAAAGTTCTGTATTATAACAGTCTCAAAAGGATCTCTTACTCCAATCCAAAATGCTGTTATGTGTAACTCTGTTTCTTAGCT | 80 | 82 |
| *SLCO1B1* | chr12:21197273-21197372 | TGCAGCAACTCTTTCTGGATCTGTAAAAGAGGTGAGGACCAATGGCAATCGACTGCCTTCAAGATTGGAGAGACAAATAGGTGATTACAAAGTGTCACAG | 87 | 84 |

# Supplementary Table S2. Taqman assay primer and probe sequences, or proprietary design

| **Primer** | **Forward (5'-3')** | **Reverse (5'-3')** | **Probe (5'-3')** |
| --- | --- | --- | --- |
| *ABR-a* | CAACTGGTAGAGCTGGGATTT | CCACCCACCTTAACTCTTCTTT | ACCTACCGCTCTGTTGCTTTCTCA |
| *ABR-b* | TTACTTTCCCGAAACCTCTCTC | CCAGACGAGATGAAGGAACTAC | TTGCAAACCCTCCTGGATCACACA |
| *APRT* | GAATGTTACCCATCACCTACCC | CCTAGGAACACACAGCTAAGAAG | CTGCTTCCAGGTCAGAGTGCAGAC |
| *C9orf140* | AACCCATCGATGCTGTTGC | CCGCTGGTGCTGGAGTA | AGGGTCCCAGTGTCACCAGAAC |
| *CPSF1* | TGGATGGCTTTGGCTTCTT | TGGTACAAGGTGTGGGTTTC | ACGGAAGTTGATGTTGTGAGGGACC |
| *GTF2H2* | ACCCAGGCTGTAGTGAAATG | GGAGGCTGAGGTACAAGAATG | TGCAACCTTTACCTCCTGGGTTCA |
| *NAALADL2* | ACATAACCTACAGTGAGGGAAATTA | AAGGAACTGGTGGGAGTAGA | TGAGACCCAAGCTTGATGTTCCCA |
| *OR2A1* | CTGAGGACAGACAGGATTTCAC | CCCATGTGGTTCTCATCCTAAG | ATTTCATGAGGCCCAGAGAAGGGC |
| *PRKG1* | GCTGTTAGGCTGATGGGTTT | GCAGAAAGGTCTTCTTCATGGT | AGGCATTGATGCTTTACTCTTGCATCTAGT |
| *PSG5* | CAGTAAGGCCTGCCCAATAA | AGAGAAGCTCAGCCCTAGAA | TGGAGCAAGGATTTAGGGACAGAGGT |
| *PTPRD-a* | GGGCTTCTGGGAGGTAATTAAG | CATACAGCAGAAGACAGAGGTG | TGAAGGTGGAGCCCTAATGAATGGG |
| *PTPRD-b* | CCATTAGCATTTCATGCTCTTTGA | CAACTGACCTTCCCTCACTATTT | TGGGAAAGTATCTATCTGTGACTCTTCTTG |
| *RAB43* | AGGCAGAGTGATGGGATTTG | CTGGATTGAGGATGTGAGGAAG | CGATCAGCAGCTGCACAATGTTGG |
| *RER1* | GTACTTCTGGTACCGTGTTGAG | CTTAGGCCTGAGCGCTTATT | TGGGATTCTCAGACTGTGGACAGGA |
| *UAP1L1* | GCTACACAGGATGTGCTTCT | ATTACTGGTTCGTCTGCTAAGG | TCTGGTTCTGCATCTGTAGGCTGC |
| **Primer** | **Assay design (Life Technologies)** |  |  |
| *CYP2A7* | Hs07545277_cn |  |  |
| *DACH1* | Hs04425852_cn |  |  |
| *ELP4* | Hs05228780_cn |  |  |
| *EPHA3* | Hs03484443_cn |  |  |
| *NAIP* | Hs03569298_cn |  |  |
| *PTPRK* | Hs06796501_cn |  |  |
| *UGT2A1* | Hs04874559_cn |  |  |

# Supplementary Table S3. Genotype and phenotype of study cohort

(Shown in a separate document)

# Supplementary Table S4. Number of CNVs of each type by calling algorithm

| **Algorithm** | **Copy number call** | | | | **Total Identified** | **Only identified by other algorithm** | **% identified by algorithm** |
| --- | --- | --- | --- | --- | --- | --- | --- |
|  | **0** | **1** | **3** | **4** |  |  |  |
| **CNVPartition** | 6205 | 12553 | 5262 | 1309 | 25329 | 35564 | 42% |
| **PennCNV** | 5848 | 31457 | 16012 | 995 | 54312 | 6581 | 89% |
| **GNOSIS** | 3979 | 12926 | 4526 | 13 | 21444 | 39449 | 35% |
| **QuantiSNP** | 8789 | 30653 | 10740 | 6978 | 57160 | 3733 | 94% |

# Supplementary Table S5. Nanostring validation results of predicted copy number deletions associated with breast cancer risk across eight gene loci in 48 study samples.

| **Sample** | ***ZNF385B*** | ***CALCRL*** | ***TFPI*** | ***GTF2H2*** | ***SLCO1B1*** | ***FGFR1OP2*** | ***TM7SF3*** | ***ALX1*** |
| --- | --- | --- | --- | --- | --- | --- | --- | --- |
| **1** | 1.00 | 2.00 | 2.00 | 2.00 | 2.00 | 2.00 | 2.00 | 2.00 |
| **2** | 2.07 | 1.91 | 1.66 | 1.48 | 1.98 | 1.98 | 1.84 | 2.23 |
| **3** | 0.98 | 1.99 | 1.60 | 1.93 | 1.94 | 2.08 | 2.08 | 1.95 |
| **4** | 0.89 | 1.97 | 1.82 | 2.19 | 1.94 | 2.03 | 2.22 | 1.79 |
| **5** | 2.05 | 2.13 | 2.16 | 1.88 | 2.07 | 2.05 | 1.92 | 2.28 |
| **6** | 1.01 | 2.05 | 1.80 | 1.64 | 1.95 | 1.95 | 1.86 | 2.12 |
| **7** | 2.18 | 2.11 | 2.01 | 1.83 | 2.39 | 2.07 | 1.90 | 2.56 |
| **8** | 1.99 | 1.72 | 1.44 | 2.51 | 1.99 | 2.41 | 2.15 | 1.99 |
| **9** | 0.87 | 1.85 | 1.59 | 1.88 | 1.87 | 2.10 | 1.98 | 2.02 |
| **10** | 1.88 | 1.89 | 1.52 | 1.92 | 1.92 | 2.22 | 2.00 | 2.13 |
| **11** | 0.90 | 1.91 | 1.50 | 1.51 | 2.00 | 2.03 | 1.85 | 2.05 |
| **12** | 1.92 | 1.71 | 1.49 | 1.54 | 1.68 | 2.01 | 1.62 | 1.90 |
| **13** | 2.00 | 2.00 | 2.00 | 2.00 | 2.00 | 2.00 | 2.00 | 2.00 |
| **14** | 2.14 | 1.71 | 1.99 | 2.53 | 2.42 | 1.94 | 1.95 | 2.06 |
| **15** | 2.17 | 1.71 | 1.67 | 1.88 | 2.06 | 2.24 | 2.11 | 1.82 |
| **16** | 2.04 | 2.03 | 2.11 | 1.54 | 2.21 | 1.84 | 1.92 | 2.05 |
| **17** | 2.10 | 2.08 | 1.87 | 2.91 | 2.20 | 1.92 | 2.03 | 2.03 |
| **18** | 2.22 | 1.84 | 2.21 | 2.56 | 2.04 | 2.10 | 2.06 | 1.91 |
| **19** | 2.05 | 1.96 | 2.11 | 1.21 | 2.21 | 1.91 | 1.79 | 2.20 |
| **20** | 0.00 | 1.40 | 2.15 | 2.80 | 2.52 | 2.04 | 2.15 | 2.17 |
| **21** | 2.40 | 2.61 | 1.97 | 2.32 | 2.73 | 1.78 | 2.80 | 1.63 |
| **22** | 2.12 | 2.07 | 1.96 | 1.98 | 2.21 | 1.93 | 2.08 | 2.02 |
| **23** | 2.59 | 2.16 | 2.22 | 1.62 | 2.43 | 1.99 | 2.05 | 2.68 |
| **24** | 2.36 | 2.08 | 2.15 | 2.79 | 2.41 | 2.11 | 2.12 | 2.13 |
| **25** | 2.00 | 2.00 | 2.00 | 3.03 | 2.00 | 2.00 | 2.00 | 2.00 |
| **26** | 2.02 | 2.08 | 1.57 | 2.00 | 1.97 | 2.12 | 1.85 | 1.99 |
| **27** | 2.46 | 2.11 | 2.07 | 1.26 | 2.20 | 2.15 | 1.95 | 2.43 |
| **28** | 2.14 | 2.25 | 1.88 | 1.69 | 2.17 | 1.99 | 1.88 | 2.17 |
| **29** | 2.12 | 2.12 | 1.97 | 1.91 | 2.21 | 2.05 | 1.73 | 2.16 |
| **30** | 1.35 | 2.50 | 2.22 | 2.29 | 2.49 | 1.86 | 1.91 | 2.85 |
| **31** | 1.96 | 2.09 | 1.75 | 1.78 | 2.07 | 2.20 | 1.90 | 2.29 |
| **32** | 2.30 | 2.54 | 2.06 | 1.21 | 2.38 | 2.03 | 1.67 | 2.73 |
| **33** | 0.98 | 2.22 | 1.87 | 1.72 | 2.09 | 1.95 | 1.72 | 2.30 |
| **34** | 1.02 | 2.32 | 2.24 | 1.55 | 2.07 | 2.18 | 1.78 | 2.37 |
| **35** | 2.07 | 2.03 | 2.08 | 1.42 | 2.10 | 2.03 | 1.68 | 2.06 |
| **36** | 2.20 | 1.90 | 1.76 | 1.85 | 2.13 | 2.05 | 1.85 | 2.20 |
| **37** | 2.00 | 2.00 | 2.00 | 2.00 | 2.00 | 2.00 | 2.00 | 2.00 |
| **38** | 1.16 | 2.29 | 1.96 | 3.35 | 2.87 | 2.54 | 2.51 | 2.56 |
| **39** | 1.03 | 2.00 | 1.87 | 2.60 | 2.18 | 2.21 | 2.10 | 2.26 |
| **40** | 2.27 | 1.79 | 1.77 | 1.70 | 2.05 | 2.41 | 2.40 | 1.98 |
| **41** | 2.13 | 1.91 | 1.89 | 2.70 | 2.21 | 2.22 | 2.15 | 2.10 |
| **42** | 2.28 | 1.77 | 1.88 | 3.44 | 2.05 | 2.35 | 2.41 | 1.95 |
| **43** | 2.17 | 1.95 | 1.71 | 1.98 | 2.19 | 2.18 | 2.36 | 2.16 |
| **44** | 2.22 | 1.91 | 2.00 | 2.19 | 2.15 | 2.40 | 2.33 | 2.05 |
| **45** | 2.55 | 1.99 | 1.95 | 1.80 | 2.24 | 2.25 | 2.09 | 2.49 |
| **46** | 2.07 | 1.88 | 1.89 | 2.63 | 1.88 | 2.23 | 2.19 | 2.24 |
| **47** | 2.08 | 1.97 | 1.96 | 1.80 | 2.24 | 2.22 | 2.01 | 2.26 |
| **48** | 1.06 | 1.76 | 1.91 | 2.14 | 2.08 | 2.36 | 2.21 | 2.16 |

^a^ Test CNV from Table 1.

Colour key: Yellow – deletion predicted by calling algorithms only; Blue – deletion detected using Nanostring only; Green – deletion predicted calling algorithms and detecting using Nanostring; White – no detection predicted by calling algorithm or detected using Nanostring.

# Supplementary Table S6. Associations with breast cancer risk for deletion variants overlapping gene regions. Genes that overlap with the Human CNV Map are shown in bold.

| **Gene(s)^a^** | **Chr** | **Number of non-affected** | **Number of affected** | **Strat logRR** | **RR (95% CI)** | **P-Value** | **Q-value** | **MAF** | **Overlap Human CNV Map** |
| --- | --- | --- | --- | --- | --- | --- | --- | --- | --- |
| *FGFR1OP2* | 12 | 14 | 1 | -1.63 | 0.2 (0.1-0.38) | 0.0005 | 0.75 | 0.6 | No |
| *APBA2* | 15 | 1 | 11 | 1.31 | 3.71 (1.51-9.11) | 0.002 | 0.96 | 0.5 | No |
| *FAM189A1* | 15 | 1 | 11 | 1.31 | 3.71 (1.51-9.11) | 0.002 | 0.96 | 0.5 | No |
| *TM7SF3* | 12 | 10 | 1 | -1.6 | 0.2 (0.09-0.45) | 0.004 | 0.96 | 0.5 | No* |
| *PRPF8* | 17 | 0 | 8 | 1.51 | 4.53 (1.58-12.9) | 0.004 | 0.96 | 0.3 | No |
| *PABPC4L* | 4 | 1 | 10 | 1.28 | 3.58 (1.35-9.46) | 0.005 | 0.96 | 0.5 | No |
| *CALCRL* | 2 | 0 | 8 | 1.42 | 4.13 (1.29-13.2) | 0.006 | 0.96 | 0.3 | No* |
| *TFPI* | 2 | 0 | 8 | 1.42 | 4.13 (1.29-13.2) | 0.006 | 0.96 | 0.3 | No* |
| ***GTF2H2C*** | **5** | **48** | **31** | **-0.44** | 0.64 (0.45-0.91) | **0.013** | 0.96 | **3.4** | **Yes** |
| ***GTF2H2*** | **5** | **48** | **31** | **-0.44** | 0.64 (0.45-0.91) | **0.013** | 0.96 | **3.4** | **Yes** |
| *PLIN3* | 19 | 0 | 6 | 1.64 | 5.14 (1.71-15.5) | 0.014 | 0.96 | 0.3 | No |
| *PYCRL* | 8 | 10 | 3 | -0.92 | 0.4 (0.15-1.05) | 0.018 | 0.96 | 0.6 | No |
| *TIGD5* | 8 | 10 | 3 | -0.92 | 0.4 (0.15-1.05) | 0.018 | 0.96 | 0.6 | No |
| *RILP* | 17 | 1 | 9 | 1.14 | 3.11 (1.23-7.89) | 0.019 | 0.96 | 0.4 | No |
| *CPSF1* | 8 | 8 | 17 | 0.71 | 2.03 (1.09-3.81) | 0.02 | 0.96 | 1.1 | No* |
| *ENTPD2* | 9 | 17 | 4 | -0.79 | 0.45 (0.24-0.86) | 0.023 | 0.96 | 0.9 | No |
| *NPDC1* | 9 | 17 | 4 | -0.79 | 0.45 (0.24-0.86) | 0.023 | 0.96 | 0.9 | No |
| *NTNG2* | 9 | 0 | 5 | 1.57 | 4.82 (1.37-16.9) | 0.024 | 0.96 | 0.2 | No |
| *C9orf139* | 9 | 17 | 4 | -0.79 | 0.45 (0.24-0.87) | 0.024 | 0.96 | 0.9 | No |
| *CACNA1B* | 9 | 14 | 6 | -0.87 | 0.42 (0.23-0.77) | 0.025 | 0.96 | 0.9 | No* |
| *SLCO1B1* | 12 | 14 | 6 | -0.86 | 0.42 (0.23-0.78) | 0.026 | 0.96 | 0.9 | No |
| *ARRDC5* | 19 | 0 | 5 | 1.66 | 5.28 (1.62-17.3) | 0.026 | 0.96 | 0.2 | No |
| ***CYFIP1*** | **15** | **0** | **5** | **1.55** | 4.71 (1.25-17.7) | **0.027** | 0.96 | **0.2** | **Yes** |
| ***NIPA1*** | **15** | **0** | **5** | **1.55** | 4.71 (1.25-17.7) | **0.027** | 0.96 | **0.2** | **Yes** |
| ***NIPA2*** | **15** | **0** | **5** | **1.55** | 4.71 (1.25-17.7) | **0.027** | 0.96 | **0.2** | **Yes** |
| ***TUBGCP5*** | **15** | **0** | **5** | **1.55** | 4.71 (1.25-17.7) | **0.027** | 0.96 | **0.2** | **Yes** |
| *FAM53B* | 10 | 5 | 0 | -1.76 | 0.17 (0.06-0.52) | 0.028 | 0.96 | 0.2 | No |
| *THSD4* | 15 | 5 | 0 | -2.08 | 0.13 (0.04-0.37) | 0.029 | 0.96 | 0.2 | No |
| *ALX1* | 12 | 5 | 0 | -1.46 | 0.23 (0.06-0.94) | 0.03 | 0.96 | 0.2 | No |
| *TBC1D3* | 17 | 5 | 0 | -1.85 | 0.16 (0.04-0.59) | 0.031 | 0.96 | 0.2 | No |
| *TBC1D3F* | 17 | 5 | 0 | -1.85 | 0.16 (0.04-0.59) | 0.031 | 0.96 | 0.2 | No |
| *GRIN1* | 9 | 19 | 7 | -0.68 | 0.51 (0.28-0.9) | 0.033 | 0.96 | 1.1 | No |
| *LRRC26* | 9 | 19 | 7 | -0.68 | 0.51 (0.28-0.9) | 0.033 | 0.96 | 1.1 | No |
| ***SPDYE2*** | **7** | **10** | **23** | **0.55** | 1.74 (1.01-3) | **0.038** | 0.96 | **1.4** | **Yes** |
| *B3GNTL1* | 17 | 3 | 8 | 0.99 | 2.68 (1.12-6.4) | 0.039 | 0.96 | 0.5 | No* |
| *ZNF385B* | 2 | 104 | 87 | -0.23 | 0.79 (0.62-1.01) | 0.04 | 0.96 | 8.2 | No* |
| *CBLB* | 3 | 0 | 5 | 0.98 | 2.65 (0.3-23.4) | 0.041 | 0.96 | 0.2 | No |
| *COL11A1* | 1 | 10 | 3 | -0.96 | 0.38 (0.17-0.88) | 0.042 | 0.96 | 0.6 | No* |
| *FUT7* | 9 | 16 | 4 | -0.68 | 0.5 (0.25-1.02) | 0.043 | 0.96 | 0.9 | No |
| *TPK1* | 7 | 4 | 0 | -2.42 | 0.09 (0.03-0.24) | 0.044 | 0.96 | 0.2 | No* |
| *RASA3* | 13 | 1 | 6 | 1.15 | 3.16 (1.03-9.73) | 0.044 | 0.96 | 0.3 | No* |
| *UHRF1* | 19 | 0 | 4 | 1.52 | 4.57 (1.09-19.2) | 0.044 | 0.96 | 0.2 | No |
| *ABR* | 17 | 7 | 17 | 0.62 | 1.85 (0.96-3.56) | 0.045 | 0.96 | 1.0 | No* |
| *L1CAM* | X | 0 | 4 | 1.46 | 4.29 (0.96-19.2) | 0.045 | 0.96 | 0.2 | No |
| *TBC1D22A* | 22 | 0 | 4 | 1.43 | 4.17 (0.9-19.5) | 0.046 | 0.96 | 0.2 | No* |
| *DR1* | 1 | 4 | 0 | -2.08 | 0.13 (0.04-0.4) | 0.046 | 0.96 | 0.2 | No |
| *AK5* | 1 | 2 | 7 | 1.16 | 3.19 (1.25-8.17) | 0.048 | 0.96 | 0.4 | No* |
| *RER1* | 1 | 15 | 22 | 0.53 | 1.69 (1.01-2.84) | 0.049 | 0.96 | 1.6 | No |
| ***PSG5*** | **19** | **44** | **29** | **-0.35** | 0.7 (0.48-1.03) | **0.049** | 0.96 | **3.2** | **Yes** |
| ***NAALADL2*** | **3** | **76** | **105** | **0.22** | 1.25 (0.96-1.62) | **0.049** | 0.96 | **7.8** | **Yes** |
| *GRM5* | 11 | 4 | 0 | -1.85 | 0.16 (0.04-0.56) | 0.049 | 0.96 | 0.2 | No* |
| *ADAMTS2* | 5 | 4 | 0 | -1.81 | 0.16 (0.05-0.56) | 0.05 | 0.96 | 0.2 | No* |

Abbreviations: Chr, chromosome; CNV, copy number variant; MAF, minor allele frequency; RR (95%CI), relative risk (95% Confidence Interval); Strat logRR, stratified log of the relative risk (Based on the stratified test statistic and robust p-value).

* CNV regions from the Zarrei et al (2015)^2^ map overlap the gene of interest but not the CNVs called by this study.

# Supplementary Table S7. Associations with ovarian cancer risk for deletion variants overlapping gene regions. Genes that overlap with the Human CNV Map are shown in bold.

| **Gene** | **Chr** | **Number of non-affected** | **Number of affected** | **Strat logRR** | **RR( 95% CI)** | **P-Value** | **Q-value** | **MAF** | **Overlap Human CNV Map** |
| --- | --- | --- | --- | --- | --- | --- | --- | --- | --- |
| *PABPC4L* | 4 | 11 | 0 | -1.52 | 0.22 (0.01-5.06) | 0.006 | 0.80 | 0.5 | No |
| ***CYP2A7*** | **19** | **75** | **4** | **-0.69** | 0.5 (0.2-1.27) | **0.007** | **0.80** | **3.4** | **Yes** |
| ***GSTT2*** | **22** | **10** | **0** | **-0.93** | 0.39 (0-84) | **0.01** | **0.80** | **0.4** | **Yes** |
| *PTPRD* | 9 | 30 | 1 | -0.92 | 0.4 (0.1-1.56) | 0.011 | 0.80 | 1.3 | No* |
| *APBA2* | 15 | 11 | 1 | -1.12 | 0.33 (0.05-2.00) | 0.011 | 0.80 | 0.5 | No |
| *FAM189A1* | 15 | 11 | 1 | -1.12 | 0.33 (0.05-2.00) | 0.011 | 0.80 | 0.5 | No |
| *DNAL1* | 14 | 11 | 0 | -1.46 | 0.23 (0.01-6.09) | 0.011 | 0.80 | 0.5 | No |
| *CSMD3* | 8 | 10 | 0 | -1.23 | 0.29 (0.01-6.44) | 0.012 | 0.80 | 0.4 | No* |
| *ELFN1* | 7 | 7 | 0 | -1.59 | 0.2 (0.03-1.65) | 0.013 | 0.80 | 0.3 | No |
| *FUT7* | 9 | 8 | 12 | 1.41 | 4.09 (1.69-9.93) | 0.016 | 0.80 | 0.9 | No |
| *CABP7* | 22 | 2 | 8 | 2.13 | 8.41 (2.71-26.1) | 0.017 | 0.80 | 0.4 | No |
| *TRAPPC2L* | 16 | 7 | 11 | 1.54 | 4.67 (1.94-11.2) | 0.017 | 0.80 | 0.8 | No |
| *NRG3* | 10 | 6 | 0 | -1.72 | 0.18 (0.01-4.86) | 0.018 | 0.80 | 0.3 | No* |
| *CCDC48* | 3 | 16 | 2 | -1.16 | 0.31 (0.09-1.03) | 0.019 | 0.80 | 0.8 | No |
| *GP9* | 3 | 16 | 2 | -1.16 | 0.31 (0.09-1.03) | 0.019 | 0.80 | 0.8 | No |
| *NEGR1* | 1 | 9 | 0 | -1.48 | 0.23 (0.01-4.22) | 0.019 | 0.80 | 0.4 | No* |
| *ABCA3* | 16 | 6 | 0 | -1.54 | 0.21 (0.02-2.27) | 0.02 | 0.80 | 0.3 | No* |
| *DACH1* | 13 | 239 | 58 | 0.45 | 1.57 (1.11-2.23) | 0.023 | 0.80 | 12.9 | No* |
| *PICALM* | 11 | 9 | 0 | -1.61 | 0.2 (0-9.96) | 0.024 | 0.80 | 0.4 | No |
| *PAOX* | 10 | 6 | 0 | -1.37 | 0.25 (0.02-3.62) | 0.024 | 0.80 | 0.3 | No |
| ***LOC440563*** | **1** | **7** | **0** | **-1.5** | 0.22 (0.01-6.77) | **0.025** | 0.80 | **0.3** | **Yes** |
| *ZDHHC21* | 9 | 8 | 0 | -1.16 | 0.31 (0-76.6) | 0.028 | 0.80 | 0.3 | No* |
| *PTPRT* | 20 | 6 | 0 | -1.65 | 0.19 (0.01-4.25) | 0.029 | 0.80 | 0.3 | No* |
| *UAP1L1* | 9 | 12 | 12 | 1.29 | 3.62 (1.53-8.53) | 0.029 | 0.80 | 1.0 | No |
| *UGT2A1* | 4 | 5 | 0 | -1.27 | 0.28 (0-68.3) | 0.03 | 0.80 | 0.2 | No* |
| *C9orf140* | 9 | 12 | 12 | 1.28 | 3.59 (1.52-8.46) | 0.03 | 0.80 | 1.0 | No* |
| *ZMAT5* | 22 | 2 | 7 | 2.02 | 7.52 (2.26-25.0) | 0.031 | 0.80 | 0.4 | No |
| *CNOT2* | 12 | 9 | 0 | -0.98 | 0.37 (0.01-13.8) | 0.032 | 0.80 | 0.4 | No* |
| *KCNMB4* | 12 | 9 | 0 | -0.98 | 0.37 (0.01-13.8) | 0.032 | 0.80 | 0.4 | No |
| ***CNTNAP3*** | **9** | **8** | **0** | **-1.28** | 0.28 (0-20.2) | **0.032** | 0.80 | **0.3** | **Yes** |
| ***FAM75A1*** | **9** | **8** | **0** | **-1.28** | 0.28 (0-20.2) | **0.032** | 0.80 | **0.3** | **Yes** |
| ***FAM75A2*** | **9** | **8** | **0** | **-1.28** | 0.28 (0-20.2) | **0.032** | 0.80 | **0.3** | **Yes** |
| *MTG1* | 10 | 5 | 0 | -1.48 | 0.23 (0.02-3.39) | 0.033 | 0.80 | 0.2 | No* |
| *LRTM2* | 12 | 6 | 0 | -1.34 | 0.26 (0.01-4.88) | 0.033 | 0.80 | 0.3 | No |
| *CALML6* | 1 | 5 | 0 | -1.64 | 0.19 (0.02-2.21) | 0.034 | 0.80 | 0.2 | No |
| *TMEM52* | 1 | 5 | 0 | -1.64 | 0.19 (0.02-2.21) | 0.034 | 0.80 | 0.2 | No |
| *ISY1* | 3 | 38 | 6 | -0.81 | 0.45 (0.2-1) | 0.034 | 0.80 | 1.9 | No |
| *RAB43* | 3 | 38 | 6 | -0.81 | 0.45 (0.2-1) | 0.034 | 0.80 | 1.9 | No |
| *GPR17* | 2 | 7 | 0 | -1.57 | 0.21 (0.02-2.39) | 0.035 | 0.80 | 0.3 | No |
| *LIMS2* | 2 | 7 | 0 | -1.57 | 0.21 (0.02-2.39) | 0.035 | 0.80 | 0.3 | No |
| *MYO7B* | 2 | 7 | 0 | -1.57 | 0.21 (0.02-2.39) | 0.035 | 0.80 | 0.3 | No* |
| *ABCA2* | 9 | 4 | 8 | 1.54 | 4.66 (1.5-14.5) | 0.035 | 0.80 | 0.5 | No* |
| *C9orf142* | 9 | 4 | 8 | 1.54 | 4.66 (1.5-14.5) | 0.035 | 0.80 | 0.5 | No |
| *CLIC3* | 9 | 4 | 8 | 1.54 | 4.66 (1.5-14.5) | 0.035 | 0.80 | 0.5 | No |
| *LCN12* | 9 | 4 | 8 | 1.54 | 4.66 (1.5-14.5) | 0.035 | 0.80 | 0.5 | No* |
| *LCNL1* | 9 | 4 | 8 | 1.54 | 4.66 (1.5-14.5) | 0.035 | 0.80 | 0.5 | No |
| *PTGDS* | 9 | 4 | 8 | 1.54 | 4.66 (1.5-14.5) | 0.035 | 0.80 | 0.5 | No |
| *CDC5L* | 6 | 8 | 0 | -1.27 | 0.28 (0.02-3.59) | 0.035 | 0.80 | 0.3 | No |
| *CBFA2T3* | 16 | 6 | 9 | 1.45 | 4.25 (1.6-11.27) | 0.036 | 0.80 | 0.7 | No* |
| *ENTPD2* | 9 | 9 | 12 | 1.25 | 3.48 (1.47-8.2) | 0.036 | 0.80 | 0.9 | No |
| *NPDC1* | 9 | 9 | 12 | 1.25 | 3.48 (1.47-8.2) | 0.036 | 0.80 | 0.9 | No |
| *APRT* | 16 | 12 | 14 | 1.11 | 3.04 (1.38-6.72) | 0.037 | 0.80 | 1.1 | No |
| *CDT1* | 16 | 12 | 14 | 1.11 | 3.04 (1.38-6.72) | 0.037 | 0.80 | 1.1 | No |
| *GALNS* | 16 | 12 | 14 | 1.11 | 3.04 (1.38-6.72) | 0.037 | 0.80 | 1.1 | No* |
| *UCK1* | 9 | 5 | 0 | -1.78 | 0.17 (0.02-1.52) | 0.037 | 0.80 | 0.2 | No |
| *C9orf139* | 9 | 9 | 12 | 1.23 | 3.43 (1.45-8.11) | 0.037 | 0.80 | 0.9 | No |
| ***CHRFAM7A*** | **15** | **10** | **0** | **-1.58** | 0.21 (0.03-1.42) | **0.04** | 0.80 | **0.4** | **Yes** |
| *C22orf25* | 22 | 7 | 1 | -1.02 | 0.36 (0.03-4.5) | 0.04 | 0.80 | 0.3 | No* |
| *FAM5C* | 1 | 6 | 0 | -0.83 | 0.44 (0-217) | 0.04 | 0.80 | 0.3 | No* |
| *NDUFS1* | 2 | 8 | 0 | -2.19 | 0.11 (0.01-2.02) | 0.042 | 0.80 | 0.3 | No* |
| *GNAO1* | 16 | 3 | 7 | 1.4 | 4.05 (1.04-15.86) | 0.042 | 0.80 | 0.4 | No |
| ***KCP*** | **7** | **15** | **3** | **-0.84** | 0.43 (0.11-1.62) | **0.044** | 0.80 | **0.8** | **Yes** |
| ***DEAF1*** | **11** | **10** | **11** | **1.09** | 2.98 (1.1-8.08) | **0.044** | 0.80 | **0.9** | **Yes** |
| *MSH4* | 1 | 7 | 0 | -0.84 | 0.43 (0-739) | 0.044 | 0.80 | 0.3 | No |
| *PTPRK* | 6 | 16 | 0 | -0.8 | 0.45 (0.02-12.25) | 0.045 | 0.80 | 0.7 | No* |
| *PRKG1* | 10 | 46 | 5 | -0.72 | 0.49 (0.21-1.16) | 0.047 | 0.80 | 2.2 | No* |
| *L1CAM* | X | 4 | 0 | -1.41 | 0.24 (0.01-5.87) | 0.048 | 0.80 | 0.2 | No |
| ***CTAGE4*** | **7** | **20** | **8** | **1.38** | 3.97 (1.7-9.29) | **0.049** | 0.80 | **1.2** | **Yes** |
| ***OR2A1*** | **7** | **20** | **8** | **1.38** | 3.97 (1.7-9.29) | **0.049** | 0.80 | **1.2** | **Yes** |
| ***OR2A42*** | **7** | **20** | **8** | **1.38** | 3.97 (1.7-9.29) | **0.049** | 0.80 | **1.2** | **Yes** |
| ***OR2A7*** | **7** | **20** | **8** | **1.38** | 3.97 (1.7-9.29) | **0.049** | 0.80 | **1.2** | **Yes** |
| *KIAA1257* | 3 | 9 | 1 | -1.22 | 0.29 (0.06-1.42) | 0.05 | 0.80 | 0.4 | No |

Abbreviations: Chr, chromosome; CNV, copy number variant; MAF, minor allele frequency; RR (95%CI), relative risk (95% Confidence Interval).; Strat logRR, stratified log of the relative risk (Based on the stratified test statistic and robust p-value).

* CNV regions from the Zarrei et al (2015)^2^ map overlap the gene of interest but not the CNVs called by this study.

## REFERENCES

1. Antoniou AC, Wang X, Fredericksen ZS, et al. A locus on 19p13 modifies risk of breast cancer in BRCA1 mutation carriers and is associated with hormone receptor-negative breast cancer in the general population. Nat Genet 2010;**42**(10):885-92.

2. Zarrei M, MacDonald JR, Merico D, et al. A copy number variation map of the human genome. Nat Rev Genet 2015;**16**(3):172-83.
